# Combining Immune Checkpoint Inhibitors with Loco-Regional Treatments in Hepatocellular Carcinoma: Ready for Prime Time?

**DOI:** 10.3390/curroncol31060242

**Published:** 2024-05-31

**Authors:** Juliette Boilève, Valentine Guimas, Arthur David, Clément Bailly, Yann Touchefeu

**Affiliations:** 1 Inserm CIC 1413, Hépato-Gastroentérologie, Institut des Maladies de l’Appareil Digestif (IMAD), CHU Nantes, Nantes Université, 44000 Nantes, France; juliette.boileve@chu-nantes.fr; 2 Department of Radiation Oncology, Institut de Cancérologie de l’Ouest, Bd Professeur Jacques Monod, 44800 Saint-Herblain, France; valentine.guimas@ico.unicancer.fr; 3 Department of Radiology, University Hospital, Nantes 1 Place Alexis Ricordeau, 44093 Nantes, France; arthur.david@chu-nantes.fr; 4 Nuclear Medicine Department, University Hospital, 44093 Nantes, France; clement.bailly@chu-nantes.fr

**Keywords:** hepatocellular carcinoma, locoregional treatment, adjuvant therapy, radio-frequency ablation, trans-arterial chemoembolization, selective internal radiation therapy, immune checkpoint inhibitors

## Abstract

Hepatocellular carcinoma (HCC) is a disease with a poor prognosis, often diagnosed at an advanced stage. Therapeutic options have developed considerably in recent years, particularly with trans-arterial treatments. Systemic treatments have also evolved significantly, with the rise of immune checkpoint inhibitors (ICI) as first-line treatment for advanced HCC. The combination of loco-regional treatments and ICI is opening up new prospects and is the subject of numerous clinical trials. Recently, two global phase 3 trials investigating ICI-based adjuvant combinations have demonstrated improvements in recurrence-free survival or progression-free survival in patients treated with resection, ablation, or trans-arterial chemoembolization. However, mature data and overall survival results are still awaited but will be difficult to interpret. We are at the start of a new era of combinations of loco-regional treatments and immunotherapy. The identification of the best therapeutic strategies and predictive biomarkers is a crucial issue for future standards in clinical practice.

## 1. Introduction

Primary liver cancer is the seventh most frequently occurring cancer in the world and the second most common cause of cancer mortality. Hepatocellular carcinoma (HCC) represents approximately 75% of these cancers [1,2].

Staging of HCC is important to determine the optimal therapy. The BCLC (Barcelona Clinic Liver Cancer) staging system [3] identifies patients with early HCC who may benefit from resection or local ablation (stage 0 and A), patients with intermediate (stage B) or advanced stage (stage C) who may benefit from intra-arterial or systemic treatments, and those with a very poor life expectancy (stage D) [4]. Liver transplantation gives the possibility of curing both the tumor and the underlying liver disease. The Milan criteria (one lesion < 5 cm or ≤three lesions < 3 cm) are currently the gold standard for selecting patients with HCC for liver transplantation [5]. For early-stage HCC, surgical resection or local ablation are recommended. Trans-arterial chemoembolization (TACE) or selective internal radiation therapy (SIRT) (=trans arterial radioembolization TARE) are alternative options. For intermediate-stage HCC, loco-regional therapies such as TACE or SIRT can be discussed. Stereotactic body radiotherapy (SBRT) is also an alternative option, in particular in the case of a high risk of local failure after thermal ablation due to location [6]. The eligibility criteria for these treatments vary by location, in particular between Europe and North America, Japan, Korea, and China [7].

Systemic therapies are recommended for patients with advanced HCC (BCLC-C) or BCLC-B tumors after trans arterial therapies have failed or are contraindicated. In recent years, interest in immune checkpoint inhibitors (ICI) has grown rapidly, and they have become the standard treatment for advanced HCC. Combinations of atezolizumab plus bevacizumab, camrelizumab plus rivoceranib, or tremelimumab plus durvalumab are now recommended as first-line treatment [8,9,10].

Combination treatments could reduce the risk of local or distant recurrences. Several trials have investigated the combination of locoregional treatments and tyrosine kinase inhibitors (TKI), with negative results (Table 1). The combination of locoregional treatments and ICI opens up new perspectives and is the subject of clinical trials, in order to take advantage of their potential synergistic effects. The aim of this review is to explain the different types of local treatments and to analyze the results of studies that have combined local treatments with immunotherapy.

## 2. Local and Locoregional Therapies

Approximately 30% of patients are initially treated by resection or local ablation, but the disease recurrence rate after resection or local ablation is significant and ranges from 50% to 70% at 5 years [7]. Thermal ablation by radio-frequency ablation (RFA) or micro-wave ablation (MWA) is recommended in very early-stage disease (BCLC 0) and early-stage disease (BCLC A). In very early-stage disease, with tumors < 2 cm diameter, RFA has demonstrated similar outcomes to liver resection, with lower morbidity compared to surgery. In patients with early-stage HCC (up to three lesions ≤ 3 cm), ablation or resection should be discussed in multidisciplinary meetings, with location and size of tumors, portal hypertension, and comorbidities making the decision [6].

The almost exclusive arterial vascularization of HCC has led to the emergence of trans-arterial treatments, involving intra-arterial infusion of chemotherapy alone, mixed with a lipiodol contrast agent that is selectively retained by the HCC nodules, embolization material, or microspheres loaded with radionuclides. Trans-arterial treatments are proposed for patients in the intermediate stage (BCLC B). For patients at BCLC 0 or BCLC A stages, if ablation or resection is not possible, TACE or SIRT may be considered [3]. TACE has been studied in randomized trials in comparison with the best supportive care, and only two studies found a survival advantage for the TACE group in selected patients with preserved liver function and early intermediate BCLC A or BCLC B stage [12,13]. TACE can be performed conventionally using lipiodol, which is standard practice, or using doxorubicin-eluting beads (DEB)—TACE. SIRT is based on the injection of microspheres loaded with a radionuclide, most frequently ^90^Y, into the hepatic arterial circulation and has no ischemic effect. ^166^Ho is a high-energy β-emitting isotope with a shorter half-life than ^90^Y (27 h versus 64 h). The safety of ^166^Ho SIRT was confirmed in HCC in the HEPAR Primary Study, with less than 10% unacceptable toxicity [14]. The LEGACY study is a retrospective study that included 162 patients treated with ^90^Y-loaded resin microspheres. It showed clinically meaningful response rates, with an overall response rate (ORR) of 88.3% and prolonged duration of response rates (RR) > 6 months in the treatment with SIRT of unresectable solitary HCC ≤ 8 cm [15]. SARAH and SIRveNIB trials investigating using Y-90 resin microspheres [16,17] did not show an improvement in overall survival (OS) compared to sorafenib. However, personalized dosimetry, now part of the standard procedure, was not used at the time of these trials. A secondary analysis of data from the SARAH trial showed that higher tumor radiation–absorbed dose computed at technetium 99m macroaggregated human albumin SPECT/CT was associated with better OS and disease control in HCC treated with SIRT than sorafenib [18]. In phase 3 studies, SIRT was associated with higher ORR, delayed tumor progression in the liver, and fewer adverse events than sorafenib [19,20,21]. DOSISPHERE-01 is a randomized phase 2 study, which included 60 patients with at least one measurable lesion of 7 cm or more in size. Patients were randomized to receive yttrium-90-loaded glass microspheres according to standard or personalized dosimetry. The long-term median OS was 24.8 months in the personalized dosimetry group versus 10.7 months in the standard dosimetry group [22,23]. In BCLC, the place of SIRT is based on the results of LEGACY, which is a non-randomized study that only included single HCC of less than 8 cm, whereas the data from DOSISPHERE-01 also showed promising results in patients with a larger tumor burden, at least one measurable lesion of 7 cm or more in size. At last, a meta-analysis of studies comparing TACE and SIRT was performed, with data suggesting that SIRT provides a significantly longer time to progression than TACE, although the two treatments do not significantly differ in terms of OS [24]. Similarly, a phase 2 prospective study showed a superior tumor control and survival with SIRT, compared with TACE, in selected participants with early and intermediate HCC [25]. Another study evaluated an adjuvant selective infusion of ^166^Ho-microspheres after RFA for the treatment of HCC and showed that it can be administered safely at a dose of 90 Gy to the treatment volume while reaching a dose of ≥120 Gy to the target volume. It may be a favorable adjuvant therapy for HCC lesions 2–5 cm [26].

Early data on radiotherapy for HCC were disappointing, mainly because of the toxicity, but stereotactic body radiation therapy (SBRT) optimizes tumor targeting and spares non-tumor tissue. SBRT provides a 2-year local control of 90% to 95% and is an effective noninvasive treatment option for patients with limited disease [27]. In a recent meta-analysis of 14 studies and 2974 patients comparing RFA or SBRT, SBRT was associated with higher rate of complete response and better local tumor control, particularly for tumors larger than 2 cm and for locations difficult for thermal ablation (sub-diaphragmatic, close to vessels) [28]. In a recently published randomized phase 2 study (TRENDY trial), which included 30 patients with HCC with one to three lesions (maximum total diameter < 6 cm), the median time to progression was 19 months in the SBRT arm versus 12 months in the DEB-TACE arm, and the median time to local control was up to 40 versus 12 months [29]. Three related toxicity grades ≥3 were observed after DEB-TACE, but none after SBRT.

## 3. Immune Checkpoint Inhibitors

Treatment of advanced HCC is based on systemic therapies. Initially, sorafenib was the only validated first-line treatment for HCC, based on the SHARP and the Asia-Pacific studies. The SHARP study, which included 602 patients, showed a median OS of 10.7 months in the sorafenib group and 7.9 months in the placebo group [30]. The Asia-Pacific study, which included 271 patients, showed a median OS of 6.5 months in the sorafenib arm, compared with 4.2 months in the placebo arm [31]. Lenvatinib, a multi-TKI that targets VEGFR (Vascular Endothelial Growth Factor Receptors) 1–3, FGFR (Fibroblast Growth Factor Receptors) 1–4, PDGF (Platelet-derived Growth Factor) receptor α, RET (Rearranged During Transfection), and KIT (receptor tyrosine kinase), was also an option in first line in some countries, based on the REFLECT study, which included 954 patients, and showed a median survival time for lenvatinib of 13.6 months vs. 12.3 months for sorafenib, meeting criteria for non-inferiority [32]. Several trials have evaluated immunotherapy as a single agent, with negative results. For example, the CheckMate 459 trial showed a better safety profile for nivolumab as a single agent compared with sorafenib, but no significant OS [33]. Combination trials have therefore been developed (combination with anti-VEGR or dual ICIs). Since the Imbrave150 trial [34], immunotherapy has become a first-line treatment for HCC. Imbrave150 was a phase 3 trial comparing the combination of atezolizumab (anti-programmed cell death ligand-1—anti-PDL1) and bevacizumab (anti VEGF) with sorafenib, which included 668 patients. OS at 12 months was 67.2% (95% CI, 61.3 to 73.1) with atezolizumab and bevacizumab and 54.6% (95% CI, 45.2 to 64.0) with sorafenib. Another phase 3 study, CARES-310, was performed comparing camrelizumab (anti-programmed cell death-1—anti-PD1) plus rivoceranib (anti-VEGFR2) versus sorafenib and showed a statistically significant and clinically meaningful benefit in progression-free survival (PFS) and OS [9]. The HIMALAYA trial [10] included 1171 patients and compared the association of tremelimumab (anti-cytotoxic T lymphocyte-associated antigen 4—anti-CTLA4) and durvalumab (anti-PDL1) (STRIDE) to sorafenib. The median OS was 16.43 months (95% CI, 14.16 to 19.58) with STRIDE, 16.56 months (95% CI, 14.06 to 19.12) with durvalumab, and 13.77 months (95% CI, 12.25 to 16.13) with sorafenib. The four-year results OS rate remained higher for STRIDE at 25.2%, versus 15.1% for sorafenib [10]. Two phase 3 studies have combined immunotherapy with TKI, with negative results. The COSMIC-312 trial compared cabozantinib plus atezolizumab versus sorafenib: median PFS was superior in the combination treatment group but median OS was not superior [35]. The LEAP-002 study compared pembrolizumab plus lenvatinib to lenvatinib plus placebo, and the combination did not significantly improved OS and PFS [36]. In second line, KEYNOTE 240 showed a median OS of 14.6 months for pembrolizumab versus 13.0 months for placebo and is approved by the FDA [37].

There is no validated predictive biomarker reported so far. The liver immune microenvironment displays a population of immunosuppressive cells, notably liver resident macrophages, known as Kupffer cells, regulatory T cells, monocyte-derived macrophages, and myeloid-derived suppressor cells, conferring to the liver tolerogenic properties. Genomic profiling of more than 700 tumors has enabled the identification of HCC (25% of cases) expressing markers of an inflammatory response (immune class), with two subclasses characterized by adaptive or exhausted immune responses [38]. A more recently published analysis integrating RNA and whole-exome sequencing, TCR sequencing, multiplex immunofluorescence, and immunohistochemistry could categorize inflamed (around 35% of cases) and non-inflamed tumors. The inflamed category can be divided into three components: the previously described immune active and exhausted subclasses and an immune-like subclass [39]. These classifications are promising predictive biomarkers, but validation in prospective studies is needed.

Combining locoregional treatments with ICI is also under investigation, aiming at improving clinical outcomes, notably reducing the risk of HCC recurrence, and improving response rate and survivals.

## 4. Combining Locoregional Treatments with Immune Checkpoint Inhibitors

### 4.1. Radiofrequency Ablation and Immunotherapy

Main published controlled trials assessing intra-arterial therapies and immunotherapy in HCC are presented in Table 2. Several studies have shown that ablative therapy can help stimulate antigen-specific CD4+ and CD8+ T cells in HCC patients and to active natural killer (NK) cell responses [11]. Thermal ablation induces various biological effects independent of tumor antigen release, including induction of proinflammatory cytokines (interleukin (IL)-1β, IL-6, IL-8, and Tumor Necrosis Factor) [40] and increased levels of HSP70 (heat shock proteins), a stress-induced protein. Adjuvant immunotherapy stimulates anti-tumor immunity against micrometastases after removal of the primary tumor, while neoadjuvant immunotherapy uses the primary tumor as a source of antigens to stimulate these responses. When the primary tumor is present (neoadjuvant setting), immunotherapy can promote the de novo induction of T cell-mediated immunity, the expansion of pre-existing anti-tumor T cells, and the development of a more diverse repertoire of tumor-specific T cells, more effectively than after tumor removal (adjuvant setting) [7]. There are a few clinical trials combining radiofrequency ablation (RFA) and adjuvant immunotherapy. A Chinese study has been conducted combining RFA and cellular immunotherapy in patients. The results showed a higher PFS in the RFA and cellular immunotherapy group than in the RFA alone group [41]. Another Chinese randomized trial evaluated the efficacy of the combination of radioimmunoconjugate (^131^I) metuximab and RFA compared with RFA alone. The results showed a median time to overall tumor recurrence of 17 months in the combined group and 10 months in the RFA group [42]. A phase 3 Korean randomized controlled trial showed that patients who underwent curative treatment for HCC increased recurrence-free and OS with adjuvant immunotherapy with activated CIK cells (CD3 +/CD56 + and CD3 +/CD56—T cells and CD3-/CD56 + natural killer cells) [43]. The team then conducted a 5-year follow-up, which confirmed a significant improvement in recurrence-free survival and OS in patients receiving adjuvant immunotherapy [44]. A retrospective study published in 2021 aimed to evaluate whether combined therapy with PD-1 blockade and RFA is superior to RFA monotherapy for recurrent HCC. The 1-year recurrence-free survival rate was significantly higher in the anti-PD-1 plus RFA group than in RFA alone (32.5% and 10.0%, respectively), after propensity score matching [45]. The IMMULAB study, a phase 2 trial, investigated peri-interventional treatment with pembrolizumab combined with RFA or combined with TACE and RFA in early-stage HCC with maintained liver function (Child Pugh A). The study included 30 patients. The overall response rate was 13.3%, with 6.7% complete responses and 6.7% partial responses after two cycles of pembrolizumab and before local ablation. This study did not meet its primary endpoint because the hypothesized ORR of 30% before local therapy was not reached [46].

IMBRAVE-050 is the first positive phase 3 study combining local treatments with adjuvant immunotherapy and investigating the efficacy of adjuvant atezolizumab plus bevacizumab during 17 cycles (12 months) versus active surveillance in patients with high-risk surgically resected or ablated HCC. Six hundred and sixty-eight patients were randomly assigned. At the pre-specified interim analysis, median duration of follow-up was 17.4 months. Adjuvant atezolizumab plus bevacizumab was associated with significantly improved recurrence-free survival compared with active surveillance (medians not evaluable; hazard ratio 0.72, *p* = 0.012). The median duration of treatment was 11 months for both atezolizumab and bevacizumab. Grade 3 or 4 adverse events occurred in 41% of patients who received atezolizumab plus bevacizumab and 13% of patients in the active surveillance group [8]. However, the PFS survival curves of the two groups converge after approximately 21 months of follow-up, which raises the question of the long-term effectiveness of this strategy, in particular after interruption of the adjuvant treatment. The main question is whether the treatment prevents or only delays recurrences. OS date (key-secondary endpoint) and longer follow-up are awaited. However, given the multiple modalities and lines of treatments available after recurrence, the analysis of OS data and potential benefits will be challenging.

### 4.2. SIRT and Immunotherapy

A study investigated the immune landscapes of tumor-infiltrating leucocytes (TILs), tumor tissues, and peripheral blood mononuclear cells (PBMCs) at different time points before and after SIRT for HCC. Tumors treated by SIRT had an infiltration by multiple activated immune subsets and were less immunosuppressive, compared with the TREG cells-enriched control tumors. SIRT can enhance activation and recruitment of T cells, NK cells, and NKT cells, with chemotaxis of CD8+ T cells to the tumor microenvironment (18).

#### 4.2.1. SIRT and Adjuvant Immunotherapy

A small single-center retrospective study included patients with preserved liver function (Child–Pugh score A-B7) and advanced HCC (macrovascular invasion or limited extrahepatic disease of aggressive intermediate stage) who received checkpoint inhibitor immunotherapy after SIRT. It appeared to be safe with limited treatment-related toxicity [49]. Another phase 1 prospective study showed that SIRT and Nivolumab in advanced HCC was tolerable, and that combination therapy resulted in a clinical benefit rate of 82%, with nine patients achieving stable disease [50]. A phase 2 trial evaluated the ORR in patients with Child–Pugh A cirrhosis and advanced HCC treated with nivolumab and Y90 SIRT. The study showed an ORR of 30% [51]. Another trial compared the efficacy of combined therapy and immunotherapy alone. The median OS was significantly higher in the combination group (19.8 vs. 9.5 months) [52].

#### 4.2.2. SIRT and Neodjuvant Immunotherapy

Recently, a study assessed the impact of the addition of three infusions of atezolizumab plus bevacizumab before and after SIRT on patients’ outcomes. Thirty-five HCC patients treated with SIRT were included, of whom 23% also received atezolizumab plus bevacizumab infusions. The median OS was not reached for patients who received atezolizumab plus bevacizumab in combination with SIRT and 14 months for patients only treated by SIRT. The median progression-free survival was higher in the group treated with SIRT and atezolizumab plus bevacizumab vs. SIRT alone (11.3 months vs. 5.8 months) [47]. The results of this study are promising, especially as neoadjuvant treatment could have an interesting immune effect (18). Studies with larger numbers would be interesting to conclude.

### 4.3. TACE and Immunotherapy

TACE is recommended for BCLC-B tumors. In most recent randomized trials, median OS with TACE alone ranges 26–30 months, with median PFS about 7–8 months, mRECIST objective response rate around 50%, and RECIST response rate around 30%. Combination with systemic therapies would aim at increasing these outcomes. TACE increases the release of antigens and proinflammatory cytokines and the secretion of VEGF and HIF-1a (hypoxia-inducible factor). Targeting the vascularization, in particular by blocking VEGFR 1–3, can induce immunomodulation, decreasing myeloid-derived suppressor cells, increasing dendritic cells and T cells, and increasing PD1 expression on T cells. These data pave the way to clinical trials investigations of combinations of TACE with systemic treatments. Trials investigating TACE combined with TKI have all been negative so far (Table 1) [11]. Trials studying the combination of TACE with immunotherapy have been initiated. A pilot study looked at the safety, feasibility, and efficacy of tremelimumab (anti-CTLA4) combined with TACE for patients with HCC BLCB C. The study also included BLCL C group treated with ablation (RFA or cryoablation) instead of TACE. The median time to tumor progression was 7.4 months. Median OS was 12.3 months during a follow-up of 18.8 months [53]. Another study published in 2021 evaluated the efficacy and safety of RFA and TACE, combined with postoperative cytokine induced killer (CIK) cell immunotherapy for patients with HCC. The results showed an overall survival of 42.1 ± 5.6 months in the RFA + TACE + CIK group and 37.8 ± 4.8 months in RFA + TACE group. The 5-year OS rate was 29.3% in the RFA + TACE + CIK group and 13.8% in the RFA + TACE group. In conclusion, in this study, RFA and TACE combined with postoperative autologous CIK cell reinfusion have significant efficacy in the treatment of primary HCC, which can improve the postoperative quality of life and raise the survival rate of patients, with tolerable adverse reactions [54].

EMERALD-1 is the first positive phase 3 trial combining TACE with systemic treatment, and the first results were presented at the ASCO GI meeting in January 2024. EMERALD-1 is a double-blind phase 3 study, which compared treatment with durvalumab + bevacizumab + TACE, durvalumab + TACE, or TACE in patients with HCC treatable with TACE and with preserved liver function (Child A to B7). Progression-free survival (PFS) was significantly improved for durvalumab + bevacizumab + TACE vs. TACE, with a median PFS of 15.0 vs. 8.2 months (hazard ratio 0.77; 95% confidence interval 0.61–0.98; *p* = 0.032). The secondary endpoint of PFS for durvalumab + TACE vs. TACE was not statistically significant (median PFS 10.0 vs. 8.2 months; hazard ratio, 0.94; 95% CI, 0.75–1.19; *p* = 0.638). OS was not significant in the interim analysis; EMERALD-1 is ongoing for the final analysis of overall survival. Safety was manageable and consistent with the safety profiles of durvalumab, bevacizumab and TACE. Grade 3/4 treatment-related adverse events in durvalumab + bevacizumab + TACE, durvalumab + TACE, and TACE were 32.5%, 15.1%, and 13.5%, respectively. A total of 8.4%, 4.3%, and 3.5% discontinued treatment due to a treatment-related adverse event [48]. The hazard ratio for PFS is 0.77, which may not be sufficient to predict an impact on overall survival. In a meta-analysis from 27 randomized trials testing kinase inhibitors or monoclonal antibodies, an HR of PFS < 0.6 was correlated with significant overall survival. However, most of the studies investigated TKI, and this cut-off is not validated for combinations therapies with immunotherapy. This trial also highlights the role of bevacizumab in the treatment of HCC. The was no arm combining TACE with bevacizumab alone; the benefits of durvalumab in the combination arm can thus be questioned [55,56]. Targeting the VEGF pathway in combination with immunotherapy led to the two positives trials IMBRAVE 050 (atezolizumab with bevacizumab) and CARES-310 (camrelizumab plus rivoceranib, a selective VEGFR2 inhibitor), whereas other combination of immunotherapy with TKI were negative (LEAP-02, COSMIC-312). The selective targeting of the VEGF pathway could therefore have a specific synergistic effect with immunotherapy and drive the benefits of the durvalumab–bevacizumab arm. As in the IMBRAVE-050 trial, OS results and longer follow-up are awaited, but data will also be difficult to interpret.

### 4.4. SBRT and Immunotherapy

There is a strong rationale for combining immunotherapy with SBRT [57]. Preclinical data have shown that the combination of SBRT and ICI was synergistic. Radiotherapy triggers immune activity and can switch a “cold” tumor to a hot tumor with enhanced inflammation and TILs. ICI could overcome the radiation-induced exhaustion in CD8 T cells and restore their anti-tumor immune responses [58]. ICIs could also enhance the radiation therapy-induced abscopal effect, a systemic immune response mediated by the effects of radiation on the immune system [59].

Retrospective reviews, case reports/series, and prospective data have looked at the association between SBRT and ICI in HCC specifically [60,61]. A prospective study analyzed safety of radiotherapy in patients with HCC followed by pembrolizumab and showed an acceptable toxicity [62].

A multicenter phase 1 randomized trial published in 2023 evaluated the safety and efficacy of SBRT (40 Gy 5 fractions) and ICI in advanced or unresectable HCC [63]. SBRT was delivered in 13 patients followed by nivolumab alone or nivolumab plus ipilimumab. Dose-limiting toxicities occurred in two patients (15.2%) and grade 3 hepatotoxicity occurred in four patients (30.8%) in the entire cohort. With a median follow-up of 42.7 months, clinical outcomes favored the combination ICIs with a 3-year OS of 57% (90% CI, 23–81%).

SBRT and ICI combination therapy for HCC appears to be safe and effective. However, optimal timing of ICI to SBRT (before, concurrent, or after), optimal RT dose/fractionation scheme, and patients’ selection have to be specified. There is a strong investigational interest for combination SBRT and ICI in HCC, and several prospective clinical trials registered at www.ClinicalTrials.gov are ongoing.

### 4.5. Future Studies

Currently, multiple different combination therapies are being studied. With the emergence of multiple tyrosine kinase inhibitors along with immunotherapy, the future perspective is focusing on finding combination therapies (Table 3). For example, LEAP-012 is a phase 3 evaluating the efficacy and safety of lenvatinib and pembrolizumab in combination with TACE versus TACE plus placebo in participants with incurable, non-metastatic HCC. EMERALD-2, the results of a phase 3, randomized, double-blind, placebo-controlled study of durvalumab monotherapy or in combination with bevacizumab as adjuvant therapy in patients with HCC after curative hepatic resection or ablation are expected (NCT03847428). EMERALD-3 is an ongoing randomized phase 3, open-label study assessing the efficacy and safety of durvalumab and tremelimumab, with or without lenvatinib, given concurrently with TACE vs. TACE alone in patients with intermediate-stage HCC not amenable to curative therapy (NCT05301842). AB-LATE 02 is an ongoing study, investigating neoadjuvant atezolizumab and adjuvant atezolizumab + bevacizumab in combination with percutaneous RFA (NCT04727307). Another phase 2 study, ROWAN, is assessing the safety and efficacy of SIRT administered before initiation of durvalumab with tremelimumab in HCC patients who are not candidates for resection, thermal ablation, or liver transplant (NCT05063565). Finally, a trial is evaluating the added value of ^166^Holmium SIRT to atezolizumab plus bevacizumab in patients with non-resectable HCC (NCT05705791). The results of these trials will be available in the next few years.

## 5. Conclusions

HCC is a disease with a poor prognosis, as it is often diagnosed at an advanced stage. Therapeutic options have developed a lot in recent years, especially with trans-arterial treatments. Similarly, systemic treatments have evolved significantly, with the rise of immunotherapy in the first line of advanced HCC. The possibility of combining local treatments and immunotherapy is the subject of many studies. At present, the results of two global positive phase 3 studies (IMBRAVE-050 and EMERALD-1) have been presented with improvements of recurrence-free survival or PFS. Longer follow-up and OS data are awaited. However, OS data will be challenging to interpret, considering the multiple modalities and lines of treatment available after progression. If there is no improvement in OS, the integration in guidelines and reimbursement policies could vary among continents and countries. The identification and the best neo(adjuvant) strategies with predicative biomarkers remains the main challenge for future clinical practice.

## Figures and Tables

**Table 1:** Randomized controlled trials assessing intra-arterial therapies and tyrosine kinase inhibitors in HCC, reprinted from Ref. [11].

Randomized Controlled Trial	Region	Experimental Arms	Primary End Point	Outcomes
**Chemoembolization**				
Kudo et al., 2011 (POST-TACE trial)	Japan, South Korea	cTACE (responders) plus sorafenib (n = 229) vs. cTACE plus placebo (n = 229)	TTP	5.4 months vs. 3.7 months; HR 0.87 (95% CI 0.70–1.09); *p* = 0.252
Kudo et al., 2014 (BRISK-TA trial)	Global	cTACE or DEB-TACE plus brivanib (n = 249) vs. cTACE plus placebo (n = 253)	OS	26.4 months vs. 26.1 months; HR 0.90 (95% CI 0.66–1.23); *p* = 0.53
Lencioni et al., 2016 (SPACE trial)	Global	DEB-TACE plus sorafenib (n = 154) vs. DEB-TACE plus placebo (n = 153)	TTP	5.6 months vs. 5.5 months; HR 0.797 (95% CI 0.588–1.080); *p* = 0.072
Meyer et al., 2017 (TACE 2 trial)	UK	DEB-TACE plus sorafenib (n = 157) vs. DEB-TACE plus placebo (n = 156)	PFS	7.8 months vs. 7.7 months; HR 1.03 (95% CI 0.75–1.42); *p* = 0.85
Kudo et al., 2018 (ORIENTAL trial)	Japan, South Korea, Taiwan	cTACE plus orantinib (n = 445) vs. cTACE plus placebo (n = 444)	OS	31.1 months vs. 32.3 months; HR 1.090 (95% CI 0.878–1.352); *p* = 0.435
Kudo et al., 2019 (TACTICS trial)	Japan	cTACE plus sorafenib (n = 80) vs. cTACE (n = 76)	mPFS	25.2 months vs. 13.5 months; HR 0.59 (95% CI 0.41–0.87); *p* = 0.006
Park et al., 2019 (STAH trial)	South Korea	cTACE plus sorafenib (n = 170) vs. sorafenib (n = 169)	OS	12.8 months vs. 10.8 months; HR 0.91 (CI 0.69–1.21); *p* = 0.290
**Transarterial radioembolization**				
Ricke et al., 2019 (SORAMIC trial)	Europe, Turkey	TARE plus sorafenib (n = 216) vs. sorafenib (n = 208)	OS	12.1 months vs. 11.4 months; HR 1.01 (95% CI 0.81–1.25); *p* = 0.95

Abbreviations: CI: confidence interval; cTACE: conventional transarterial chemoembolization; DEB-TACE: drug-eluting bead transarterial chemoembolization; HR; hazard ratio; OS: overall survival; PFS: progression-free survival; TARE; transarterial radio embolization; TTP: time to progression.

**Table 2:** Randomized controlled trials assessing loco-regional therapies and immunotherapy in HCC.

Randomized Controlled Trial	Experimental Arms	Primary End Point	Outcomes
**Radiofrequency ablation and immunotherapy**			
Bian et al. [42]	RFA followed by radioimmunoconjugate (131I) metuximab (n = 62) vs. RFA alone (n = 65)	Overall Tumor Recurrence	17 months vs. 10 months; HR = 0.60, 95% CI = 0.38 to 0.96, *p* = 0.03
Qin et al. (IMBRAVE-050) [8]	Adjuvant atezolizumab + bevacizumab (n = 334) vs. active surveillance in surgically resected or ablated HCC (n = 334)	RFS	medians, not evaluable; hazard ratio, 0.72 adjusted 95% CI 0.53–0.98; *p* = 0.012
**SIRT and immunotherapy**			
Mejait et al. [47]	3 infusions of atezolizumab + bevacizumab before and after SIRT (n = 8) vs. SIRT alone (n = 27)	mOS	Not reached vs. 14 months
**TACE and immunotherapy**			
EMERALD-1 [48]	Durvalumab + bevacizumab + TACE vs. durvalumab + TACE vs. TACE	mPFS	Median PFS 10.0 vs. 8.2 months; hazard ratio, 0.94; 95% CI, 0.75–1.19; *p* = 0.638

Abbreviations: CI: confidence interval; TACE: transarterial chemoembolization; HR; hazard ratio; OS: overall survival; PFS: progression-free survival; RFA: radiofrequency ablation; RFS: recurrence-free survival; TARE; transarterial radio embolization; TTP: time to progression.

**Table 3:** Main ongoing phase 3 trials combining local or trans-arterial therapies for hepatocellular carcinoma.

Acronyme	Arms	Primary End Point	Clinical Trial Registration
**Combination with locoregional therapies**			
TACE-3	Nivolumab plus DEB-TACE vs. DEB-TACE	OS	NCT04268888
LEAP-012	Lenvatinib plus pembrolizumab plus cTACE vs. cTACE	PFS–OS	NCT04246177
CheckMate 74W	Nivolumab plus ipilimumab plus cTACE vs. nivolumab plus placebo plus cTACE cTACE plus placebo	TTTP–OS	NCT04340193
EMERALD-3	TACE with durvalumab and tremelimumab, with or without lenvatinib vs. TACE	PFS	NCT05301842
**Adjuvant treatment (after resection or ablation)**			
EMERALD-2	Durvalumab +/− bevacizumab vs. placebo	RFS	NCT03847428
JUPITER 04	Torpalimab vs. placebo	RFS	NCT03859128
KEYNOTE-937	Pembrolizumab vs. placebo	RFS–OS	NCT03867084
CheckMate 9DX	Nivolumab vs. placebo	RFS	NCT03383458

Abbreviations: cTACE: conventional transarterial chemoembolization; DEB-TACE: drug-eluting bead transarterial chemoembolization; OS: overall survival; PFS: progression-free survival; RFS: recurrence-free survival; TTTP: time to TACE progression.

## References

[1] McGlynn K.A., Petrick J.L., El-Serag H.B. (2021). Epidemiology of Hepatocellular Carcinoma. Hepatology.

[2] Sung H., Ferlay J., Siegel R.L., Laversanne M., Soerjomataram I., Jemal A., Bray F. (2021). Global Cancer Statistics 2020: GLOBOCAN Estimates of Incidence and Mortality Worldwide for 36 Cancers in 185 Countries. CA Cancer J. Clin..

[3] Reig M., Forner A., Rimola J., Ferrer-Fábrega J., Burrel M., Garcia-Criado Á., Kelley R.K., Galle P.R., Mazzaferro V., Salem R. (2022). BCLC strategy for prognosis prediction and treatment recommendation: The 2022 update. J. Hepatol..

[4] Forner A., Reig M., Bruix J. (2018). Hepatocellular carcinoma. Lancet.

[5] Clavien P.-A., Lesurtel M., Bossuyt P.M.M., Gores G.J., Langer B., Perrier A. (2012). Recommendations for liver transplantation for hepatocellular carcinoma: An international consensus conference report. Lancet Oncol..

[6] Vogel A., Cervantes A., Chau I., Daniele B., Llovet J.M., Meyer T., Nault J.-C., Neumann U., Ricke J., Sangro B. (2018). Hepatocellular carcinoma: ESMO Clinical Practice Guidelines for diagnosis, treatment and follow-up. Ann. Oncol..

[7] Llovet J.M., Pinyol R., Yarchoan M., Singal A.G., Marron T.U., Schwartz M., Pikarsky E., Kudo M., Finn R.S. (2024). Adjuvant and neoadjuvant immunotherapies in hepatocellular carcinoma. Nat. Rev. Clin. Oncol..

[8] Qin S., Chen M., Cheng A.-L., Kaseb A.O., Kudo M., Lee H.C., Yopp A.C., Zhou J., Wang L., Wen X. (2023). Atezolizumab plus bevacizumab versus active surveillance in patients with resected or ablated high-risk hepatocellular carcinoma (IMbrave050): A randomised, open-label, multicentre, phase 3 trial. Lancet.

[9] Qin S., Chan S.L., Gu S., Bai Y., Ren Z., Lin X., Chen Z., Jia W., Jin Y., Guo Y. (2023). Camrelizumab plus rivoceranib versus sorafenib as first-line therapy for unresectable hepatocellular carcinoma (CARES-310): A randomised, open-label, international phase 3 study. Lancet.

[10] Sangro B., Chan S.L., Kelley R.K., Lau G., Kudo M., Sukeepaisarnjaroen W., Yarchoan M., De Toni E.N., Furuse J., Kang Y.K. (2024). Four-year overall survival update from the phase III HIMALAYA study of tremelimumab plus durvalumab in unresectable hepatocellular carcinoma. Ann Oncol..

[11] Llovet J.M., De Baere T., Kulik L., Haber P.K., Greten T.F., Meyer T., Lencioni R. (2021). Locoregional therapies in the era of molecular and immune treatments for hepatocellular carcinoma. Nat. Rev. Gastroenterol. Hepatol..

[12] Llovet J.M., Real M.I., Montaña X., Planas R., Coll S., Aponte J., Ayuso C., Sala M., Muchart J., Solà R. (2002). Arterial embolisation or chemoembolisation versus symptomatic treatment in patients with unresectable hepatocellular carcinoma: A randomised controlled trial. Lancet.

[13] Lo C.-M., Ngan H., Tso W.-K., Liu C.-L., Lam C.-M., Poon R.T.-P., Fan S.-T., Wong J. (2002). Randomized controlled trial of transarterial lipiodol chemoembolization for unresectable hepatocellular carcinoma. Hepatology.

[14] Reinders M.T.M., van Erpecum K.J., Smits M.L.J., Braat A.J.A.T., de Bruijne J., Bruijnen R., Sprengers D., de Man R.A., Vegt E., IJzermans J.N.M. (2022). Safety and Efficacy of 166Ho Radioembolization in Hepatocellular Carcinoma: The HEPAR Primary Study. J. Nucl. Med..

[15] Salem R., Johnson G.E., Kim E., Riaz A., Bishay V., Boucher E., Fowers K., Lewandowski R., Padia S.A. (2021). Yttrium-90 Radioembolization for the Treatment of Solitary, Unresectable HCC: The LEGACY Study. Hepatology.

[16] Vilgrain V., Pereira H., Assenat E., Guiu B., Ilonca A.D., Pageaux G.-P., Sibert A., Bouattour M., Lebtahi R., Allaham W. (2017). Efficacy and safety of selective internal radiotherapy with yttrium-90 resin microspheres compared with sorafenib in locally advanced and inoperable hepatocellular carcinoma (SARAH): An open-label randomised controlled phase 3 trial. Lancet Oncol..

[17] Chow P.K., Gandhi M., Tan S.B., Khin M.W., Khasbazar A., Ong J., Choo S.P., Cheow P.C., Chotipanich C., Lim K. (2018). SIRveNIB: Selective Internal Radiation Therapy Versus Sorafenib in Asia-Pacific Patients with Hepatocellular Carcinoma. J. Clin. Oncol..

[18] Hermann A.-L., Dieudonné A., Ronot M., Sanchez M., Pereira H., Chatellier G., Garin E., Castera L., Lebtahi R., Vilgrain V. (2020). Relationship of Tumor Radiation–absorbed Dose to Survival and Response in Hepatocellular Carcinoma Treated with Transarterial Radioembolization with 90Y in the SARAH Study. Radiology.

[19] Salem R., Gordon A.C., Mouli S., Hickey R., Kallini J., Gabr A., Mulcahy M.F., Baker T., Abecassis M., Miller F. (2016). Y90 Radioembolization Significantly Prolongs Time to Progression Compared with Chemoembolization in Patients with Hepatocellular Carcinoma. Gastroenterology.

[20] Salem R., Lewandowski R.J., Kulik L., Wang E., Riaz A., Ryu R.K., Sato K.T., Gupta R., Nikolaidis P., Miller F.H. (2011). Radioembolization results in longer time-to-progression and reduced toxicity compared with chemoembolization in patients with hepatocellular carcinoma. Gastroenterology.

[21] Riaz A., Gabr A., Abouchaleh N., Ali R., Al Asadi A., Mora R., Kulik L., Desai K., Thornburg B., Mouli S. (2018). Radioembolization for hepatocellular carcinoma: Statistical confirmation of improved survival in responders by landmark analyses. Hepatology.

[22] Garin E., Tselikas L., Guiu B., Chalaye J., Edeline J., de Baere T., Assénat E., Tacher V., Robert C., Terroir-Cassou-Mounat M. (2021). Personalised versus standard dosimetry approach of selective internal radiation therapy in patients with locally advanced hepatocellular carcinoma (DOSISPHERE-01): A randomised, multicentre, open-label phase 2 trial. Lancet Gastroenterol. Hepatol..

[23] Garin E., Tselikas L., Guiu B., Chalaye J., Rolland Y., de Baere T., Assenat E., Tacher V., Palard X., Déandreis D. (2024). Long-Term Overall Survival After Selective Internal Radiation Therapy for Locally Advanced Hepatocellular Carcinomas: Updated Analysis of DOSISPHERE-01 Trial. J. Nucl. Med..

[24] Brown A.M., Kassab I., Massani M., Townsend W., Singal A.G., Soydal C., Moreno-Luna L., Roberts L.R., Chen V.L., Parikh N.D. (2022). TACE versus TARE for patients with hepatocellular carcinoma: Overall and individual patient level meta analysis. Cancer Med..

[25] Dhondt E., Lambert B., Hermie L., Huyck L., Vanlangenhove P., Geerts A., Verhelst X., Aerts M., Vanlander A., Berrevoet F. (2022). 90Y Radioembolization versus Drug-eluting Bead Chemoembolization for Unresectable Hepatocellular Carcinoma: Results from the TRACE Phase II Randomized Controlled Trial. Radiology.

[26] Hendriks P., Rietbergen D.D.D., van Erkel A.R., Coenraad M.J., Arntz M.J., Bennink R.J., Braat A.E., Crobach S., van Delden O.M., Dibbets-Schneider P. (2024). Adjuvant holmium-166 radioembolization after radiofrequency ablation in early-stage hepatocellular carcinoma patients: A dose-finding study (HORA EST HCC trial). Eur. J. Nucl. Med. Mol. Imaging.

[27] Schaub S.K., Hartvigson P.E., Lock M.I., Høyer M., Brunner T.B., Cardenes H.R., Dawson L.A., Kim E.Y., Mayr N.A., Lo S.S. (2018). Stereotactic Body Radiation Therapy for Hepatocellular Carcinoma: Current Trends and Controversies. Technol. Cancer Res. Treat..

[28] Rim C.H., Lee J.S., Kim S.Y., Seong J. (2022). Comparison of radiofrequency ablation and ablative external radiotherapy for the treatment of intrahepatic malignancies: A hybrid meta-analysis. JHEP Rep..

[29] Méndez Romero A., Van Der Holt B., Willemssen F.E.J.A., De Man R.A., Heijmen B.J.M., Habraken S., Westerveld H., Van Delden O.M., Klümpen H.-J., Tjwa E.T.T.L. (2023). Transarterial Chemoembolization with Drug-Eluting Beads Versus Stereotactic Body Radiation Therapy for Hepatocellular Carcinoma: Outcomes from a Multicenter, Randomized, Phase 2 Trial (the TRENDY Trial). Int. J. Radiat. Oncol. Biol. Phys..

[30] Llovet J.M., Ricci S., Mazzaferro V., Hilgard P., Gane E., Blanc J.-F., de Oliveira A.C., Santoro A., Raoul J.-L., Forner A. (2008). Sorafenib in advanced hepatocellular carcinoma. N. Engl. J. Med..

[31] Cheng A.-L., Kang Y.-K., Chen Z., Tsao C.-J., Qin S., Kim J.S., Luo R., Feng J., Ye S., Yang T.-S. (2009). Efficacy and safety of sorafenib in patients in the Asia-Pacific region with advanced hepatocellular carcinoma: A phase III randomised, double-blind, placebo-controlled trial. Lancet Oncol..

[32] Kudo M., Finn R.S., Qin S., Han K.-H., Ikeda K., Piscaglia F., Baron A., Park J.-W., Han G., Jassem J. (2018). Lenvatinib versus sorafenib in first-line treatment of patients with unresectable hepatocellular carcinoma: A randomised phase 3 non-inferiority trial. Lancet.

[33] Yau T., Park J.-W., Finn R.S., Cheng A.-L., Mathurin P., Edeline J., Kudo M., Harding J.J., Merle P., Rosmorduc O. (2022). Nivolumab versus sorafenib in advanced hepatocellular carcinoma (CheckMate 459): A randomised, multicentre, open-label, phase 3 trial. Lancet Oncol..

[34] Finn R.S., Qin S., Ikeda M., Galle P.R., Ducreux M., Kim T.-Y., Kudo M., Breder V., Merle P., Kaseb A.O. (2020). Atezolizumab plus Bevacizumab in Unresectable Hepatocellular Carcinoma. N. Engl. J. Med..

[35] Kelley R.K., Rimassa L., Cheng A.-L., Kaseb A., Qin S., Zhu A.X., Chan S.L., Melkadze T., Sukeepaisarnjaroen W., Breder V. (2022). Cabozantinib plus atezolizumab versus sorafenib for advanced hepatocellular carcinoma (COSMIC-312): A multicentre, open-label, randomised, phase 3 trial. Lancet Oncol..

[36] Llovet J.M., Kudo M., Merle P., Meyer T., Qin S., Ikeda M., Xu R., Edeline J., Ryoo B.-Y., Ren Z. (2023). Lenvatinib plus pembrolizumab versus lenvatinib plus placebo for advanced hepatocellular carcinoma (LEAP-002): A randomised, double-blind, phase 3 trial. Lancet Oncol..

[37] Finn R.S., Ryoo B.-Y., Merle P., Kudo M., Bouattour M., Lim H.Y., Breder V., Edeline J., Chao Y., Ogasawara S. (2020). Pembrolizumab as Second-Line Therapy in Patients with Advanced Hepatocellular Carcinoma in KEYNOTE-240: A Randomized, Double-Blind, Phase III Trial. J. Clin. Oncol..

[38] Sia D., Jiao Y., Martinez-Quetglas I., Kuchuk O., Villacorta-Martin C., Castro De Moura M., Putra J., Camprecios G., Bassaganyas L., Akers N. (2017). Identification of an Immune-specific Class of Hepatocellular Carcinoma, Based on Molecular Features. Gastroenterology.

[39] Montironi C., Castet F., Haber P.K., Pinyol R., Torres-Martin M., Torrens L., Mesropian A., Wang H., Puigvehi M., Maeda M. (2023). Inflamed and non-inflamed classes of HCC: A revised immunogenomic classification. Gut.

[40] Chu K.F., Dupuy D.E. (2014). Thermal ablation of tumours: Biological mechanisms and advances in therapy. Nat. Rev. Cancer.

[41] Cui J., Wang N., Zhao H., Jin H., Wang G., Niu C., Terunuma H., He H., Li W. (2014). Combination of radiofrequency ablation and sequential cellular immunotherapy improves progression-free survival for patients with hepatocellular carcinoma. Int. J. Cancer.

[42] Bian H., Zheng J.-S., Nan G., Li R., Chen C., Hu C.-X., Zhang Y., Sun B., Wang X.-L., Cui S.-C. (2014). Randomized Trial of [^131^I] Metuximab in Treatment of Hepatocellular Carcinoma after Percutaneous Radiofrequency Ablation. JNCI J. Natl. Cancer Inst..

[43] Lee J.H., Lee J.-H., Lim Y.-S., Yeon J.E., Song T.-J., Yu S.J., Gwak G.-Y., Kim K.M., Kim Y.J., Lee J.W. (2015). Adjuvant immunotherapy with autologous cytokine-induced killer cells for hepatocellular carcinoma. Gastroenterology.

[44] Lee J.-H., Lee J.H., Lim Y.-S., Yeon J.E., Song T.-J., Yu S.J., Gwak G.-Y., Kim K.M., Kim Y.J., Lee J.W. (2019). Sustained efficacy of adjuvant immunotherapy with cytokine-induced killer cells for hepatocellular carcinoma: An extended 5-year follow-up. Cancer Immunol. Immunother..

[45] Wang X., Liu G., Chen S., Bi H., Xia F., Feng K., Ma K., Ni B. (2021). Combination therapy with PD-1 blockade and radiofrequency ablation for recurrent hepatocellular carcinoma: A propensity score matching analysis. Int. J. Hyperth..

[46] Vogel A., Waidmann O., Müller T., Siegler G.M., Goetze T.O., De Toni E.N., Gonzalez-Carmona M.A., Hausner G., Geissler M., Fischer von Weikersthal L. (2023). IMMULAB: A phase II trial of immunotherapy with pembrolizumab in combination with local ablation for patients with early-stage hepatocellular carcinoma (HCC). J. Clin. Oncol..

[47] Mejait A., Roux C., Soret M., Larrey E., Wagner M., Bijot J.C., Lussey-Lepoutre C., Thabut D., Goumard C., Maksud P. (2024). Enhanced therapeutic outcomes with atezolizumab-bevacizumab and SIRT combination compared to SIRT alone in unresectable HCC: A promising approach for improved survival. Clin. Res. Hepatol. Gastroenterol..

[48] Lencioni R., Kudo M., Erinjeri J., Qin S., Ren Z., Chan S., Arai Y., Heo J., Mai A., Escobar J. (2024). EMERALD-1: A phase 3, randomized, placebo-controlled study of transarterial chemoembolization combined with durvalumab with or without bevacizumab in participants with unresectable hepatocellular carcinoma eligible for embolization. J. Clin. Oncol..

[49] Zhan C., Ruohoniemi D., Shanbhogue K.P., Wei J., Welling T.H., Gu P., Park J.S., Dagher N.N., Taslakian B., Hickey R.M. (2020). Safety of Combined Yttrium-90 Radioembolization and Immune Checkpoint Inhibitor Immunotherapy for Hepatocellular Carcinoma. J. Vasc. Interv. Radiol..

[50] Fenton S.E., Kircher S.M., Mulcahy M.F., Mahalingam D., Salem R., Lewandowski R., Kulik L., Benson A.B., Kalyan A. (2021). A phase I study of nivolumab (NIVO) in combination with TheraSphere (Yttrium-90) in patients with advanced hepatocellular cancer. J. Clin. Oncol..

[51] Tai D., Loke K., Gogna A., Kaya N.A., Tan S.H., Hennedige T., Ng D., Irani F., Lee J., Lim J.Q. (2021). Radioembolisation with Y90-resin microspheres followed by nivolumab for advanced hepatocellular carcinoma (CA 209-678): A single arm, single centre, phase 2 trial. Lancet Gastroenterol. Hepatol..

[52] Yeo Y.H., Liang J., Lauzon M., Luu M., Noureddin M., Ayoub W., Kuo A., Sankar K., Gong J., Hendifar A. (2023). Immunotherapy and Transarterial Radioembolization Combination Treatment for Advanced Hepatocellular Carcinoma. Off. J. Am. Coll. Gastroenterol. ACG.

[53] Duffy A.G., Ulahannan S.V., Makorova-Rusher O., Rahma O., Wedemeyer H., Pratt D., Davis J.L., Hughes M.S., Heller T., ElGindi M. (2017). Tremelimumab in combination with ablation in patients with advanced hepatocellular carcinoma. J. Hepatol..

[54] Ji Q., Fu Y., Zhu X., Wang L., Ling C. (2021). Effect of RFA and TACE combined with postoperative cytokine-induced killer cell immunotherapy in primary hepatocellular carcinoma. J. BUON.

[55] Llovet J.M., Montal R., Villanueva A. (2019). Randomized trials and endpoints in advanced HCC: Role of PFS as a surrogate of survival. J. Hepatol..

[56] Llovet J.M., Villanueva A., Marrero J.A., Schwartz M., Meyer T., Galle P.R., Lencioni R., Greten T.F., Kudo M., Mandrekar S.J. (2021). Trial Design and Endpoints in Hepatocellular Carcinoma: AASLD Consensus Conference. Hepatology.

[57] Lee Y.H., Tai D., Yip C., Choo S.P., Chew V. (2020). Combinational Immunotherapy for Hepatocellular Carcinoma: Radiotherapy, Immune Checkpoint Blockade and Beyond. Front. Immunol..

[58] Chakraborty M., Abrams S.I., Camphausen K., Liu K., Scott T., Coleman C.N., Hodge J.W. (2003). Irradiation of tumor cells up-regulates Fas and enhances CTL lytic activity and CTL adoptive immunotherapy. J. Immunol..

[59] Liu Y., Dong Y., Kong L., Shi F., Zhu H., Yu J. (2018). Abscopal effect of radiotherapy combined with immune checkpoint inhibitors. J. Hematol. Oncol..

[60] Jiang J., Diaz D.A., Nuguru S.P., Mittra A., Manne A. (2022). Stereotactic Body Radiation Therapy (SBRT) Plus Immune Checkpoint Inhibitors (ICI) in Hepatocellular Carcinoma and Cholangiocarcinoma. Cancers.

[61] Munker S., Rossler D., Öcal O., Ben-Khaled N., Bernhart K., Ye L., Piseddu I., Vielhauer J., Reiter F.P., Rodriguez I. (2023). Concomitant Irradiation to Checkpoint Inhibitor Therapy of Hepatocellular Carcinoma Patients: A Systematic Retrospective, Single-Center Analysis. Oncol. Res. Treat..

[62] Luke J.J., Lemons J.M., Karrison T.G., Pitroda S.P., Melotek J.M., Zha Y., Al-Hallaq H.A., Arina A., Khodarev N.N., Janisch L. (2018). Safety and Clinical Activity of Pembrolizumab and Multisite Stereotactic Body Radiotherapy in Patients with Advanced Solid Tumors. J. Clin. Oncol..

[63] Juloori A., Katipally R.R., Lemons J.M., Singh A.K., Iyer R., Robbins J.R., George B., Hall W.A., Pitroda S.P., Arif F. (2023). Phase 1 Randomized Trial of Stereotactic Body Radiation Therapy Followed by Nivolumab plus Ipilimumab or Nivolumab Alone in Advanced/Unresectable Hepatocellular Carcinoma. Int. J. Radiat. Oncol. Biol. Phys..

